# Recent progress in enhancing built-in electric fields of perovskite solar cells via junction engineering

**DOI:** 10.3762/bjnano.17.42

**Published:** 2026-05-07

**Authors:** Tong Xiao, Ke Xu

**Affiliations:** 1 School of Electrical & Control Engineering, Shenyang Jianzhu University, Shenyang, China

**Keywords:** band alignment, built-in electric field, carrier dynamics, junction, perovskite solar cells

## Abstract

The performance of perovskite solar cells (PSCs) is primarily governed by the built-in electric field (BEF), which dictates photocarrier separation, drift-diffusion transport, and extraction pathways, thereby shaping critical device parameters such as open-circuit voltage and fill factor. This review highlights recent progress in junction engineering strategies for BEF enhancement. Homojunctions extend interfacial potential fields into the bulk through controlled doping and orientation modulation, thereby suppressing nonradiative recombination and improving carrier extraction. Gradient junctions introduce continuous compositional or bandgap tilts across the film thickness, enabling depth-extended electric fields and improved spatial continuity of charge transport. By contrast, 2D/3D heterojunctions exploit interfacial dipole formation, intrinsic polarization, and phase-penetration effects to amplify and homogenize the BEF, while simultaneously improving energy-level alignment and defect passivation. We systematically compare these strategies within a unified framework of BEF descriptors, magnitude, penetration depth, directionality, and spatial continuity. Special emphasis is placed on the potential for synergistic integration of multiple junction architectures and on the development of mild, process-compatible fabrication routes. Ultimately, optimizing the BEF provides a powerful lever for advancing power conversion efficiency, open-circuit voltage, and long-term operational stability in state-of-the-art PSCs, while avoiding the introduction of parasitic energy barriers.

## Introduction

Driven by the global push for carbon neutrality, next-generation high-efficiency and low-cost photovoltaic technologies have gained increasing attention. Due to their high absorption coefficients, long carrier diffusion lengths, tunable bandgaps, and solution processability, perovskite solar cells (PSCs) have advanced significantly since their inception [1]. The power conversion efficiency (PCE) of single-junction devices has risen from 3.8% to 27.3% [2–4], highlighting their potential to approach the Shockley–Queisser limit.

In typical heterojunction solar cells, carriers migrate at the interface through diffusion and drift, gradually establishing a built-in electric field (BEF) that aligns the Fermi levels on both sides. This field is not only the primary driving force that sustains carrier separation but also a direct determinant of device photovoltaic conversion efficiency [5–7]. In PSCs, moderate enhancement of the BEF can effectively promote the separation and extraction of electron–hole pairs, reducing recombination losses and leading to higher open-circuit voltage (*V*_oc_) and fill factor (FF) [8–12]. However, excessive enhancement of the BEF may introduce additional barriers that hinder carrier extraction [13]. Achieving effective enhancement and rational optimization of the BEF through structural design has become a central research focus for improving both the efficiency and stability of PSCs [14]. Therefore, a reexamination of junction engineering from the perspective of the BEF is warranted, with particular emphasis on unifying studies of homojunctions, gradient junctions, and 2D/3D heterojunctions into a coherent framework.

Against this background, we define a “junction” as a generalized structure formed in the bulk or at the interface, characterized by band discontinuities or polarization barriers. Its primary role is to modulate the local potential distribution and establish a stable BEF. In materials such as perovskites, which are mixed ionic–electronic conductors, the junction not only determines the driving force for carrier separation but also extends the local electric field to a broader spatial region by optimizing its position, extent, and strength. Different types of junctions enhance the magnitude and uniformity of the BEF by improving band bending, suppressing recombination pathways, or introducing additional polarization fields. These effects facilitate directional transport and prolong carrier lifetimes. Therefore, the rational construction and optimization of generalized junctions to enhance the built-in electric field have become a key research direction for improving the efficiency and stability of PSCs, laying the groundwork for the present review.

Homojunctions focus on constructing continuous band bending and Fermi level gradients within the same material system using methods such as doping, surface reconstruction, or orientation induction, thereby smoothly extending the BEF from the interface into the bulk. Their advantages include a continuous potential distribution, fewer additional interfaces, and good compatibility with existing fabrication processes. In contrast, gradient junctions employ gradual variations in composition, ion concentration, dimensionality, or bandgap along the film thickness to create a continuous barrier-reducing channel across the film [15], a strategy that emphasizes longitudinal potential continuity and a “capacitive accumulation” effect, which maintains an effective driving force over a larger thickness and is often accompanied by improved crystallinity and stress release. Heterojunction systems, especially 2D/3D heterojunctions, enhance the BEF magnitude and extend its effective depth in the near-interface region through the synergistic effects of interfacial dipoles, intrinsic polarization, and penetrating low-dimensional phases, while simultaneously suppressing defects and aligning energy levels [16]. These advantages have established heterojunctions as a major research hotspot, with current efforts shifting from simple passivation to deliberate interfacial potential design [17]. Nevertheless, the impact of phase penetration depth, residual strain, and bulk inhomogeneity on field distribution remains a critical factor limiting the realization of high-efficiency devices [18].

This review aims to summarize recent advances in junction engineering that enhance the built-in electric field in terms of magnitude, effective depth, and spatial uniformity. Over the past three years, precise design and optimization of various junction structures have effectively strengthened the BEF, leading to higher PCEs. The discussion focuses on the contributions of homojunctions, gradient junctions, and 2D/3D heterojunction strategies to BEF enhancement and concludes with a summary of the main challenges and future directions in this field.

## Review

### Homojunctions

In perovskites, homojunctions are electrically non-uniform regions created by controlled doping, targeted chemical modification, or process optimization, which induce band bending and generate localized BEFs throughout the bulk of the film [19]. The resulting BEFs, extending across both bulk and surface regions, facilitate the efficient separation of photogenerated carriers and promote their directional transport to the electrodes. Furthermore, optimized interfacial energy-level alignment enhances carrier extraction and suppresses interfacial recombination [20–21]. Therefore, the formation of a strong p–n junction is an effective strategy to improve photovoltaic device performance. Nevertheless, achieving stable p-type and n-type doping in perovskites remains more difficult than in silicon because doping can introduce lattice distortions, create defects, and promote ion migration. To address these challenges, recent research has focused on precisely tuning the local potential via surface or near-surface doping and functionalization while preserving lattice continuity. Current efforts are also directed toward extending these modulations into the bulk to realize continuous potential profiles over larger length scales. The vertical gradients and thickness-dependent extensions of these effects are discussed in the following section on gradient junctions.

**Figure 1:**
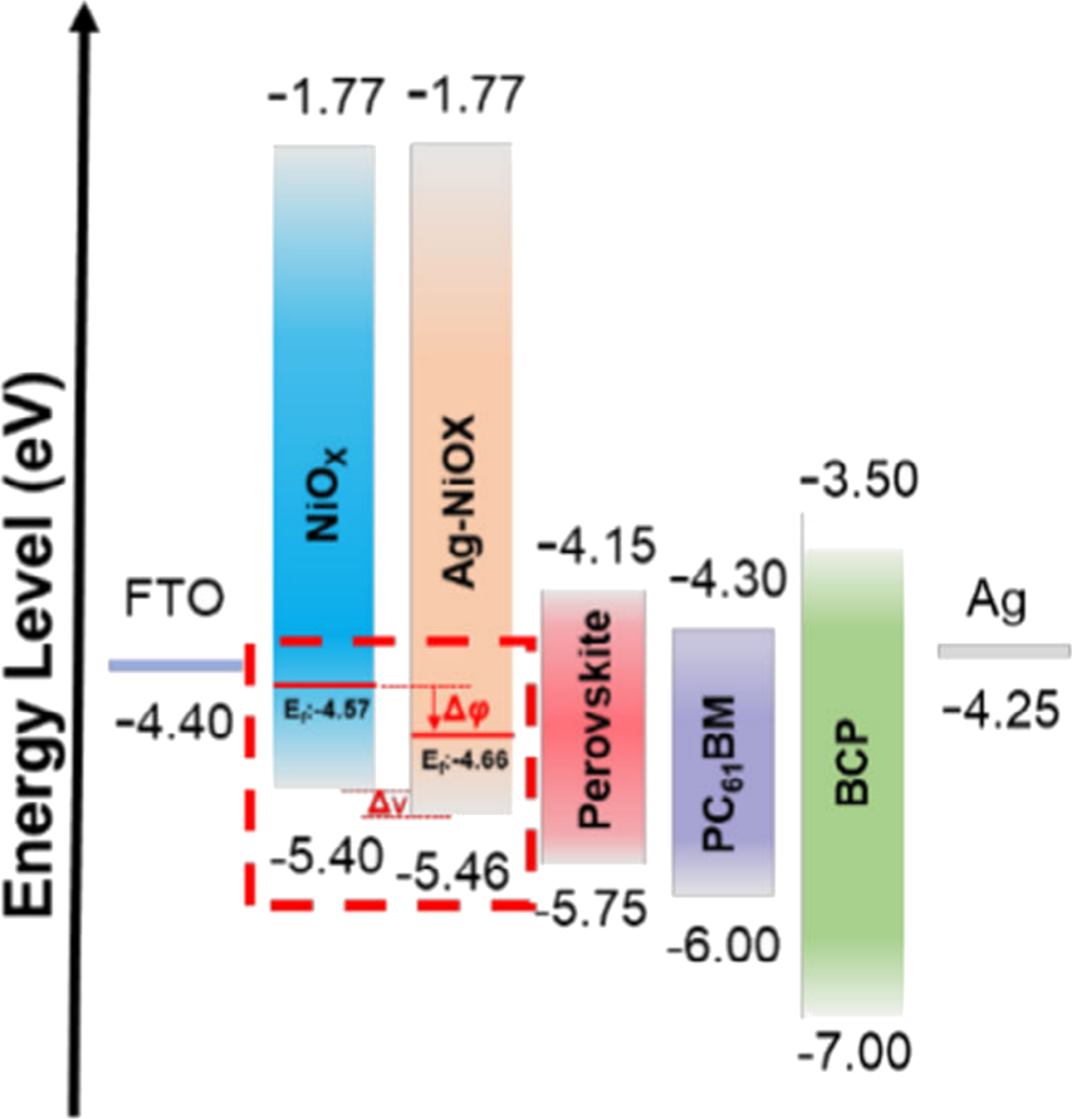
Energy levels of the hybrid NiO*_x_* hole-transporting layer in an inverted perovskite solar cell. Ag^+^ doping deepens the valence-band maximum of NiO*_x_*, inducing band bending at the NiO*_x_*/perovskite interface. This generates a localized built-in electric field that drives hole transport from the NiO*_x_* bulk toward the perovskite layer, enhancing carrier separation and suppressing interfacial recombination. Figure 1 was reproduced from [30] (© 2023 H. Wang et al. Energy & Environmental Materials published by John Wiley & Sons Australia, Ltd on behalf of Zhengzhou University, distributed under the terms of the Creative Commons Attribution 4.0 International License, https://creativecommons.org/licenses/by/4.0/).

In PSCs, the band structures of charge transport layers (CTLs) and their interfacial compatibility are critical factors governing device performance [3,22–23]. Compared with organic hole transport layers (HTLs) such as Spiro-MeOTAD [24], PEDOT:PSS [25], and PTAA [26], which are prone to moisture absorption and exhibit poor thermal stability, inorganic materials such as CuO [27] and NiO*_x_* [28–29] have attracted increasing attention due to their superior chemical stability, processability, and cost-effectiveness. Among these materials, optimization of HTL performance via single-metal or dual-metal doping has become a common strategy. For example, Wang et al. [30] constructed a NiO*_x_*/NiO*_x_* p/p^+^ homojunction by introducing Ag^+^ dopants. On the one hand, Ag^+^ doping increases the hole concentration and coordinates with lattice oxygen, thereby reducing defect states. On the other hand, it shifts the valence-band maximum (VBM) of NiO*_x_* from −5.40 eV to −5.46 eV, thereby enhancing band bending and creating a localized BEF within the HTL (as illustrated in Figure 1, showing the p/p^+^ homojunction and the directional built-in electric field for hole transport). This BEF provides a directional driving force for hole transport, enhancing carrier mobility and suppressing recombination. Additionally, it reduces trap-state capture, forming a dual-pathway mechanism that improves overall device performance. This results in better interfacial energy-level alignment and reduced interfacial recombination, increasing the PCE from 16.20% to 19.25%. Under aging conditions of 25 °C and 30% RH for 30 days, followed by 260 h of light exposure in a nitrogen atmosphere, Ag^+^ doping in sol–gel NiO*_x_* p/p^+^ homojunctions mitigated iodine vacancy formation and Pb^2+^ migration, preventing degradation pathways and reducing PCE loss from 40% to 10%.

In sequential molecular doping systems, a common problem is that dopants fail to penetrate the crystalline domains of the polymer, limiting the achievable doping level. To overcome this limitation, Zhang et al. [31] proposed an anion-assisted molecular-doping (AMD) strategy using BF_4_^−^ for the PTAA and Poly-TPD hole-transport layers in p–i–n inverted devices. The method enabled effective acceptor doping within the HTL, shifting the Fermi level downward and thereby improving alignment with the perovskite valence band. Simultaneously, interfacial interactions between BF_4_^−^ and Pb^2+^ suppressed iodine vacancies, converted the underlying perovskite from n-type to p-type, and thus induced a vertical p–n homojunction in the lower bulk region of the film. This band restructuring significantly amplified and extended the BEF, thereby facilitating directional charge separation and transport. Ultraviolet photoelectron spectroscopy (UPS) revealed an increase in the HTL work function, while capacitance measurements indicated an enhanced built-in potential. Together, these observations confirmed that AMD enhanced the BEF, reduced energy losses, and increased device efficiencies to above 24%. After 400 h of maximum power point tracking, the p–i–n perovskite solar cells optimized by BF_4_^−^ AMD retained 90.97% and 85.95% of their initial efficiencies for PTAA and Poly-TPD HTLs, respectively, demonstrating improved stability at 85 °C and high humidity.

Although doping-enhanced hole-transport layers can locally increase the BEF, this effect is largely confined to the transport layer. Shifting energy-level modulation to the buried interface of the perovskite/HTL, a critical region for carrier accumulation and recombination [32], induces band bending and the formation of a localized homojunction, which extends the BEF into the absorber layer and, to some extent, into the bulk. Liu et al. [33] demonstrated this by inserting perfluorinated copper phthalocyanine (F_16_CuPc) between the NiO*_x_*/Me-2PACz layer and perovskite, thereby shifting the locus of energy-level modulation to the buried interface. Through interfacial chemistry, a controllable potential gradient was induced across the film thickness. This induced a controllable potential gradient across the film thickness, increased the substrate work function by about 0.3 eV, and created a vertical p–n homojunction. Time-resolved photoluminescence (TRPL) measurements revealed that the carrier lifetime of the F_16_CuPc-modified device decreased from 1069.3 to 901.3 ns, indicating more efficient carrier extraction. Despite the trap density remaining nearly unchanged (2.19 × 10^15^ vs 2.00 × 10^15^ cm^−3^), EIS testing showed a significant increase in recombination resistance (from 945.1 to 5763 Ω), suggesting that the enhanced BEF through directional charge transport was the key factor in suppressing recombination. As a result, device PCE increased from 22% to 25%, and after 1100 h of aging in a nitrogen-filled glovebox without encapsulation, F_16_CuPc-induced p–n transition in the perovskite helped block iodine vacancy (PbI_2_) accumulation at grain boundary cracks, slowing efficiency degradation from 55% to 19%. Thus, BEF-induced directional charge transport is the critical mechanism behind the improved performance and stability.

Although HTL-based strategies for enhancing the BEF can, to varying degrees, extend the BEF from the transport layer to the perovskite interface, they still rely on the HTL as the platform for energy-level modulation and selective carrier transport, which limits their applicability in emerging HTL-free device architectures [34]. To address this structural limitation, Ling et al. [35] applied an isopropanol (IPA) post-treatment on finished organic–inorganic perovskite films, which induced the in situ formation of a PbI_2_-rich top layer. This created an n/n^+^ homojunction between the weakly n-type bulk and the more n-type surface layer, generating downward band bending and an additional vertical BEF that promoted carrier separation and directional transport. In HTL-free devices, this modification increased *V*_oc_ to ≈1.05 V and PCE to nearly 19%. The mechanism involves enhanced directional transport via the p–n homojunction-induced BEF, while reducing trap-state capture by lowering defect density. IPA treatment, performed in a glove box at 25 °C with <0.1 ppm H_2_O, not only formed the n/n^+^ homojunction but also suppressed iodine vacancy formation and PbI_2_ accumulation, reducing aging decay from 55% to 19% after 1100 h.

In simplified HTL-free device architectures, surface or near-surface post-treatments can form a homojunction at the top of the perovskite layer. In particular, HTL-free systems, such as those employing porous carbon electrodes, similar control can be achieved near the electrode–perovskite interface through interfacial modification. Chen et al. [36] employed a cyclopentylmethylamine (CMA) post-treatment to induce homojunction formation at the lateral microinterfaces between the carbon electrode and the perovskite. After crystallization, the perovskite within the mesoscopic framework forms numerous microcontacts with the carbon electrode. CMA infiltration from the carbon side and its interactions with Pb, I, and the organic cations not only improved local crystallinity but also induced band bending and an upward shift in the work function, a shift that partially changed the conduction type toward n^−^. As a result, an n/n^−^ homojunction formed at the microinterface, enhancing the local built-in potential (*V*_bi_) and increasing the driving force for hole injection. This effect, observed in transient photocurrent (TPC) measurements, reduced carrier extraction time from 26.91 to 9.07 μs, enhancing directional transport and reducing trap capture, which increased the PCE from 17.50% to 19.50%. The CMA post-treatment controlled the aging attenuation of carbon-based mesoporous devices at 8% after 150 days in air at 25 °C and 40% RH.

Although the aforementioned strategies optimize energy-level modulation and carrier transport at different scales, they still impose stringent requirements on fabrication uniformity, film thickness control, and precursor stoichiometry, and their process windows remain narrow, complicating large-scale manufacturing. To reconcile BEF enhancement with manufacturing feasibility, Liu et al. [37] proposed a bilayer modification strategy. They introduced natural vitamin C into the SnO_2_ ETL to reduce defects and improve electron transport. They also exploited the antioxidant and passivation effects of vitamin D_2_ to convert the perovskite surface from n-type to p-type, thereby forming a p-region approximately 80 nm thick at the top and spontaneously constructing an n–p homojunction. Accordingly, UPS and Kelvin probe force microscopy (KPFM) measurements showed that the surface work function increased by about 0.5 eV and the potential difference increased by about 0.26 V. Mott–Schottky analysis further revealed an increase in *V*_bi_ of roughly 0.1 V, indicating a significant enhancement of the bulk BEF. These results demonstrate that the bilayer modification strategy enhances the BEF while maintaining strong process compatibility, thereby providing a wider processing window for scalable device fabrication. Notably, introducing a substantial p-region at the top of the absorber extends homojunction control from the interface into the bulk, revealing a trend toward the development of continuous gradient junctions across the film thickness (see Table 1 for representative strategies and photovoltaic performance). This study quantified the impact of the n–p homojunction strategy, which approximately doubled carrier lifetime and mobility (about 17.2 cm^2^·V^−1^·s^−1^), and reduced the trap density by half, using transient photocurrent and transient photovoltage spectroscopy, and TRPL. BEF enhances directional transport through the n–p homojunction and reduces well state capture through defect passivation, remaining at 93% at room temperature for 5000 h and 85% RH/85 °C.

**Table 1:** Homojunction strategies and performance in perovskite solar cells.

Construction strategy	Device structure	BEF enhancement mechanism	p–n Region construction	PCE (%)	*V*_oc_ (V)	Ref.

doping strategies	FTO/Ag-NiO*_x_*(p^+^)/NiO*_x_*(p)/perovskite/PC_61_BM/BCP/Ag	Ag^+^ induces band bending	double-layer spin-coating	19.25	1.13	[30]
	ITO/PTAA(AMD)/FA_0.9_Cs_0.1_PbI_3_/PCBM/BCP/Ag	AMD accelerates charge transfer, reduces offset	n-to-p conversion induced homojunction	24.26	1.14	[31]
surface post-treatment	FTO/NiO*_x_*/perovskite/PCBM/BCP/Ag	PbI_2_-rich surface induces band bending	n/n^+^ surface homojunction	19.04	1.05	[35]
	FTO/compact-TiO_2_/mp-TiO_2_/mp-ZrO_2_/perovskite/mp-C	CMA induces band bending, defect passivation	n/n^−^ surface homojunction	19.50	1.02	[36]
	FTO/TiO_2_/perovskite(QDs)/carbon	QDs convert n- to p-type, increasing *V*_bi_	p–n homojunction	20.10	1.07	[34]
gradient or region construction strategies	FTO/SnO_2_-FTO/SnO_2_(VC)/perovskite(VD)/PCBM/Ag	VC assists interface, VD_2_ induces p-region	vitamin-assisted gradient doping	24.20	1.13	[37]
interface modification	ITO/NiO*_x_*/Me-2PACz/F_16_CuPc/perovskite/PC_61_BM/BCP/Ag	F_16_CuPc induces band bending	non-chemical doping	25.0	1.18	[33]
	ITO/SnO_2_/n/p-PVK-BP/spiro-OMeTAD/Ag	BP NSs induce band bending, defect passivation	spontaneous n/p homojunction	23.22	1.14	[38]

In junction engineering for high-performance PSCs, addressing both interfacial and bulk modulation during the design stage can establish a stronger internal driving field within the absorber layer, while recombination losses and improving stability are suppressed. A modification strategy based on two-dimensional black phosphorus nanosheets (BP NSs) proposed by Fan et al. [38] exemplifies this combination of interface-induced and bulk modulation. During perovskite crystallization, BP NSs, owing to their high hole mobility and elevated work function, induce a p-type region at the surface and spontaneously form n/p homojunctions with the preexisting n-type regions in the bulk, effectively creating a continuous energy-level staircase within the absorber. This structure extends the effective range of the BEF, enhances the directional charge transport and collection efficiency, and suppresses both bulk and interfacial recombination. BP NSs anchored at grain boundaries provide fast hole-transport channels, improving transport matching between the top and bottom electrodes and yielding a device efficiency of 23.2% with negligible hysteresis. Moreover, combined grain-boundary passivation and band-structure modulation by BP NSs enabled unencapsulated devices to retain over 92% efficiency maintained for 150 days (3,600 h) at 25 °C and 40% ± 10% relative humidity. Although this operational pathway shares similarities with typical 2D/3D heterojunction modifications, it is fundamentally a homojunction-based modulation that relies on electrical partitioning within the perovskite bulk.

Homojunction strategies play a key role in improving the stability of perovskite solar cells. Through doping and surface modification, they enhance the BEF, reduce interfacial defects, and prevent degradation. For example, Ag-doped NiO*_x_*/NiO*_x_* p/p^+^ homojunctions have been shown to reduce defect clusters and prevent halide ion migration, improving stability. However, challenges like phase separation and ion migration remain, with issues such as Pb–I bond rupture still persisting. To tackle these, strategies like chemical anchoring and gradient encapsulation have been suggested, such as using phosphate molecules to bond materials to the perovskite and adding thin Al_2_O_3_ layers to block water and oxygen. Understanding the role of homojunctions in BEF modulation and degradation, along with effective suppression strategies, offers solutions to improve long-term stability. Additionally, homojunctions enhance carrier dynamics by improving directional transport and reducing trap-state capture, which boosts carrier mobility and extends lifetime, key to device stability and performance.

### Gradient junctions

An ideal BEF should exhibit clear directionality and a spatially continuous potential gradient, thereby efficiently driving the directional transport of photogenerated carriers and suppressing interfacial recombination and charge accumulation characteristic of conventional flat-band architectures. To this end, the introduction of graded-junction structures into perovskite absorbers and adjacent interfacial layers, achieved by controlled through-thickness gradients in composition, ionic species or concentration, energy level or dimensionality, has recently emerged as a key strategy for strengthening the BEF. Such graded junctions establish sustained band bending aligned with the BEF through the film thickness and across interfaces, thereby markedly increasing the field strength and effective penetration depth while optimizing carrier separation, transport, and collection [39]. These processes are often accompanied by synergistic benefits, including improved crystallinity, suppression of defect states, and stress relaxation, which in turn yield concurrent gains in PCE and operational stability. Early efforts primarily employed cation or anion composition gradients to directly tailor the band structure, and subsequent research has expanded to diverse routes such as dimensional-gradient engineering, synergistic ion regulation, and tailored interfacial optimization (see Table 2 for representative graded-junction strategies and corresponding photovoltaic performance).

**Table 2:** Gradient junction strategies and performance in perovskite solar cells.

Strategy	Device structure	BEF enhancement mechanism	Gradient feature	PCE (%)	*V*_oc_ (V)	Ref.

compositional gradient	ITO/PEDOT:PSS/SnI_2_/FA_0.7_MA_0.3_Sn_0.5_Pb_0.5_I_3_/PCBM/Cu	Sn/Pb compositional tilt inducing band bending	vertical composition gradient across bulk	23.2	0.89	[40]
ionic gradient	ITO/PEDOT:PSS/Ge-doped perovskite/ICBA/Ag	Ge^2+^-induced band gradient	ionic concentration gradient	13.3	0.85	[41]
	ITO/PTAA/perovskite/C_60_/BCP/Ag	dual-ion redistribution (MA^+^ + SCN^−^)	ionic concentration gradient	22.1	0.83	[42]
	ITO/perovskite/spiro-OMeTAD/MoO_3_/Ag	band bending and recombination suppression	Rb^+^ depth gradient	21.2	1.17	[43]
	ITO/NiO*_x_*/P3CT-N/CsPbI_2.85_Br_0.15_/PCBM/BCP/Ag	energy level alignment and defect passivation	DMABr penetration gradient	20.6	1.14	[44]
	ITO/NiO*_x_*/SAM/AIV/perovskite/PC_61_BM/BCP/Cu	K^+^ slow release forming potential gradient	K^+^ release gradient	23.2	1.14	[45]
	ITO/NiO*_x_*/perovskite (p-FPEAI SGP)/C_60_/BCP/Ag	F^−^-induced molecular/ionic redistribution raising *V*_bi_	F^−^ concentration gradient	21.6	1.24	[46]
dimensional/quasi-2D gradient	ITO/PEDOT:PSS/PEA_2_(FA_0.5_MA_0.5_)_4_(Pb_0.5_Sn_0.5_)_5_I_16_/PCBM/BCP/Ag	in situ SnSe reduces traps increases *V*_bi_	Quasi-2D gradient (*n* = 3→5)	15.1	0.85	[47]
bandgap gradient	ITO/SnO_2_/PeNC/spiroOMeTAD/Au	Eg-step and ligand removal	bandgap gradient (Eg step)	16.5	1.16	[48]

In graded-junction research, directly tuning band tilting through compositional distributions is considered the most straightforward and effective strategy for enhancing the BEF. However, maintaining a continuous gradient while also ensuring structural stability and compatibility with low-temperature processing remains challenging [49–50]. Liu et al. [40] embedded SnI_2_ into the PEDOT:PSS bottom electrode and annealed the layer. During the deposition of FA_0.7_MA_0.3_Sn_0.5_Pb_0.5_I_3_, this treatment induced a vertical redistribution of Sn^2+^ and Pb^2+^, forming a compositional gradient with Sn enrichment at the bottom and Pb enrichment at the top. TOF-SIMS depth profiling and UPS measurements confirmed that this gradient introduced approximately 0.32 eV of band bending within the bulk, which generated a quasi-BEF even without external bias, raising the *V*_bi_ to 0.96 V, which promoted exciton dissociation and carrier transport. This method is compatible with low-temperature processing and maintains stability for over 1000 h. The device retained about 90% of its initial PCE after 1000 h in an unpackaged room-temperature environment with 25% humidity. The Sn/Pb gradient effectively blocks Pb diffusion to the HTL, preventing band mismatch and BEF attenuation. The gradient heterojunction maintains continuous band bending, reducing degradation and phase separation, thereby improving device stability.

Given that compositional doping can generate band gradients but often fails to simultaneously achieve bulk defect passivation and phase-order control, several studies have proposed an ionic-gradient strategy to establish a potential gradient while optimizing crystal orientation and carrier transport pathways [51]. Zhao et al. [41] introduced a gradually decreasing concentration of Ge^2+^ across the film thickness, creating a potential gradient that extends throughout the bulk of the absorber. KPFM measurements revealed a stable, uniform potential profile from the surface to the electrode, absent in the control sample. The resulting BEF provided a sustained driving force, promoting directional carrier migration, suppressing recombination, and improving charge transport and photovoltaic performance. After 250 min of continuous illumination in an N_2_ atmosphere and 3800 h of inert storage, efficiency retention rates of 95% and 85% were achieved, respectively. The enhanced stability is mainly due to the electronic compensation effect of the vertical Ge^2+^ gradient on Sn vacancies, which prevents the oxidation of Sn^2+^ to Sn^4+^ and minimizes deep-level defects. Additionally, the GE-I framework prevents ion migration and strain accumulation, suppressing failure from Sn vacancy diffusion and oxidation-induced energy level collapse, leading to longer-term stability.

In graded-junction strategies, evaporation of volatile salts combined with synergistic multi-ion interactions can both establish a potential gradient and passivate defects, thereby further enhancing the BEF. Tao et al. [42] introduced methylammonium acetate (MAAc) and methylammonium thiocyanate (MASCN) into perovskite precursors containing both Pb and Sn. MAAc volatilized rapidly, inducing surface crystallization, while SCN^−^ accumulated in the bulk and passivated deep defects. Together, these effects promoted vertically oriented, columnar crystal growth. As a result, complementary ionic gradients and an ordered, columnar grain orientation were established throughout the film thickness, producing continuous band tilting through the film thickness, which reduced carrier migration barriers, enhanced charge separation, and extraction. The stability tests demonstrated that the device retained over 90.97% of its initial efficiency after 400 h of maximum power point tracking under continuous simulated sunlight (AM 1.5G), confirming the improved stability attributed to optimized crystallization and interface passivation achieved by the BF_4_^−^ anion-assisted molecular doping strategy.

**Figure 2:**
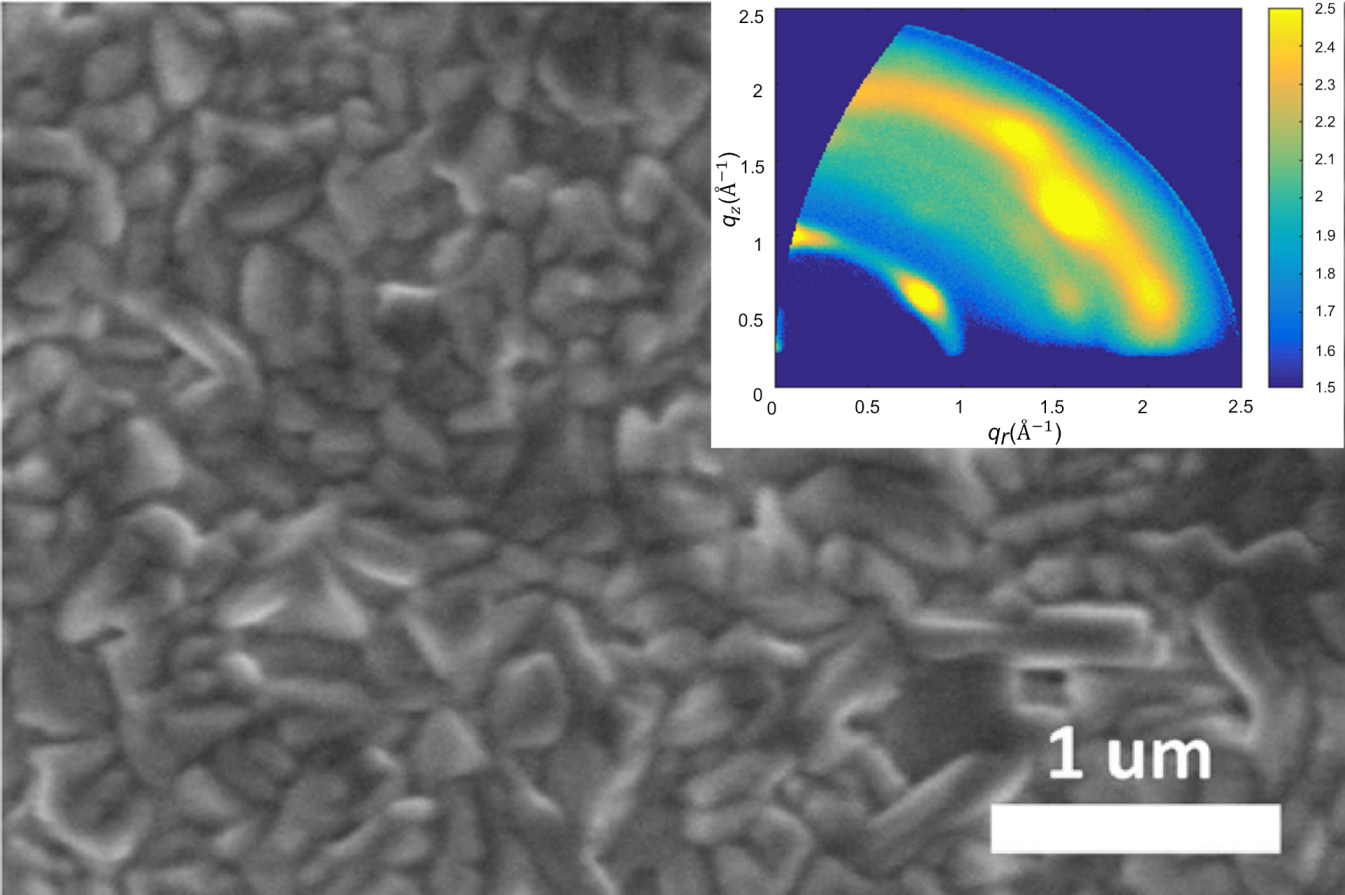
SEM and GIWAXS reveal the in situ formation of a compact and crystalline SnSe layer induced by SnAc_2_ + DMS treatment. The continuous surface coverage and distinct SnSe diffraction features confirm the presence of a hydrophobic passivation layer, which blocks moisture infiltration, suppresses interfacial degradation pathways, and thereby enhances the environmental stability of the perovskite film. Figure 2 was adapted from [47] (“In situ SnSe deposition as passivation for scalable and stable quasi-2D lead-tin perovskite solar cells”, © 2023 L. Chen et al., published by The Royal Society of Chemistry, distributed under the terms of the Creative Commons Attribution-NonCommercial 3.0 Unported License, https://creativecommons.org/licenses/by-nc/3.0/). This content is not subject to CC BY 4.0.

The graded-junction strategies, which rely on compositional and ionic modulation, typically introduce nonuniformities throughout the thickness of the film to induce band tilting. These strategies have partially improved carrier separation and defect passivation [44,52]. To address these issues, Chen et al. [47] proposed a dimensional-gradient strategy, in which an ultrathin SnSe layer is in situ deposited onto the surface of a quasi-2D Pb–Sn perovskite. This strategy induces a coordinated distribution of lower-dimensional and higher-dimensional phases along the film’s thickness. The SnSe layer interacts with the surface low-dimensional phase, suppressing its disordered aggregation and promoting the gradual extension of the high-dimensional phase toward the bottom of the film, thereby creating a smooth transition from a high-bandgap, low-dimensional phase to a low-bandgap, high-dimensional phase across the thickness of the film. The dimensional gradient, which aligns with the BEF and establishes a continuously decreasing potential barrier across the film thickness, allows carriers to migrate in an energetically favorable direction, significantly lowering migration barriers and enhancing charge-separation efficiency. SEM and GIWAXS characterization of the SnAc2 + DMS-treated perovskite film is shown in Figure 2. Accordingly, the devices demonstrated exceptional performance, with carrier lifetimes extended to 812 ns and a PCE increased to 22.75%. This underscores the potential of gradient-field engineering in 2D/3D mixed perovskite systems. Devices passivated with SnSe retained 91% of their initial efficiency after 10 days in air and showed minimal loss after a month in nitrogen or 15 h of continuous MPP tracking. This stability stems from the hydrophobicity of SnSe, which protects the active layer.

Although the aforementioned graded-junction strategies induce sustained band tilting across the film thickness and enhance carrier separation and transport, they remain insufficient for the coordinated control of interfacial defects and mechanical stress. This limitation is particularly pronounced in certain mixed-halide, wide-bandgap systems, where rapid top-down crystallization leads to accumulation and tensile stress at the film bottom, creating a gradient opposite to the BEF, which weakens the electric field and accelerates recombination and ion migration. To address this issue, Zhang et al. [45] proposed a bottom-up gradient construction strategy based on alkali-intercalated vermiculite nanosheets, abbreviated as “AIV”. At the bottom interface, AIV and the perovskite form Pb–O bonds that facilitate lattice matching and reduce stress concentration, thereby promoting more orderly crystallization. At the same time, weak interlayer van der Waals interactions allow for gradual release of K^+^ ions during growth and their penetration into the bulk, establishing a concentration gradient aligned with the BEF across the film thickness. ToF-SIMS results show pronounced K^+^ enrichment at the film bottom. This template and slow-release coupling effect produce a continuous potential descent from the bottom toward the top and a cleaner interfacial environment within the film, thereby driving directional carrier transport and suppressing recombination. AMD strategy yields a significant increase in carrier lifetime, a large improvement in hole mobility, and a reduction in trap-state density, thereby improving charge separation efficiency. After being stored in ambient air (temperature 25 °C, humidity 30–40%) for 10 days, it can still maintain an initial PCE of 91%.

Beyond establishing sustained band tilting in conventional three-dimensional perovskite films, gradient-engineering strategies also exhibit considerable potential in addressing performance bottlenecks in specific types of perovskite devices [53]. The key lies in targeting the unique limitations of various device architectures and precisely designing vertical or interfacial band gradients to optimize carrier dynamics in a customized manner [54]. Yang et al. [48] tackled charge localization and barrier-blocking issues in perovskite quantum dot (PeQD) devices caused by long-chain ligands by introducing a “reassembly process” (RP) strategy. Using a pyridine/isopropanol mixed solvent, insulating long-chain ligands were replaced with formamidinium iodide short-chain ions, enabling close-packed quantum dot assembly and vertical growth orientation. Simultaneously, layered spin-coating of nanocrystals with varying bandgaps created a top-down decreasing bandgap gradient, forming a continuous downhill potential channel along the film thickness, which synergistically mitigates charge confinement, accelerates charge extraction, and reduces interfacial impedance. As a result, the device *V*_bi_ increased from 0.92 to 0.98 V, the carrier lifetime nearly doubled, the short-circuit current density rose to 19.34 mA·cm^−2^, and the power conversion efficiency reached 16.46%, confirming the pivotal role of enhanced BEF in promoting carrier separation and collection.

Fluorinated ammonium salts can induce spatial potential gradients that are aligned with the BEF during film formation across various perovskite device systems, thereby enhancing band bending and promoting directional carrier transport [55–56]. Jiang et al. [57] introduced fluorinated organic ammonium salts in combination with organosulfur compounds into HTL-free carbon-based perovskite solar cells, resulting in a spontaneous F^−^ and S^2−^ concentration gradient at the perovskite/carbon interface, which decreases from the surface to the bulk. This gradient corresponds to a stepwise upward shift of energy levels and an increase in interfacial barrier, driving the band bending at the interface along the BEF and producing a continuous potential drop. As a result, the barrier for electron injection into the carbon electrode is reduced, bulk-to-electrode extraction is accelerated, and interfacial recombination is suppressed. Consequently, the device’s open-circuit voltage increased from 0.89 to 0.94 V, while both the short-circuit current and fill factor were improved, and the power conversion efficiency reached 20.52%, highlighting the unique contribution of ionic gradients to BEF enhancement in HTL-free architectures. They retained 79% efficiency at 85 °C for 300 h, 88% after 30 days in nitrogen, and 80.8% under 400 h of continuous illumination, with enhanced stability attributed to hydrophobic fluorinated chains, –SF_3_ defect passivation, and lattice strain release.

In graded junctions, BEF degradation arises directly from the collapse of the intentionally engineered depth-dependent potential profile. Ionic migration smooths the designed band tilt, while phase segregation introduces local band discontinuities, disrupting both the magnitude and the spatial coherence of the BEF-failure modes fundamentally more gradient-sensitive than those in homojunctions. Accordingly, stabilization must target the preservation of this depth profile: Ion-blocking layers (e.g., Al_2_O_3_) immobilize mobile species, and dimensional/interface stabilizers such as SnSe prevent low-dimensional phase dropout and band-topology disruption. SnI_2_-induced Sn/Pb gradients further suppress Pb diffusion, delaying field flattening. These stabilization routes directly correspond to the gradient-specific failure modes identified above. In contrast to the homojunction case, where field decay is mainly localized, graded junctions require preserving an extended depth profile. Thus, clarifying the degradation mechanisms enables the design of suppression strategies that are precisely matched to the spatially coupled nature of graded junctions.

### 2D/3D heterojunctions

#### Interface stabilization for BEF continuity

**Figure 3:**
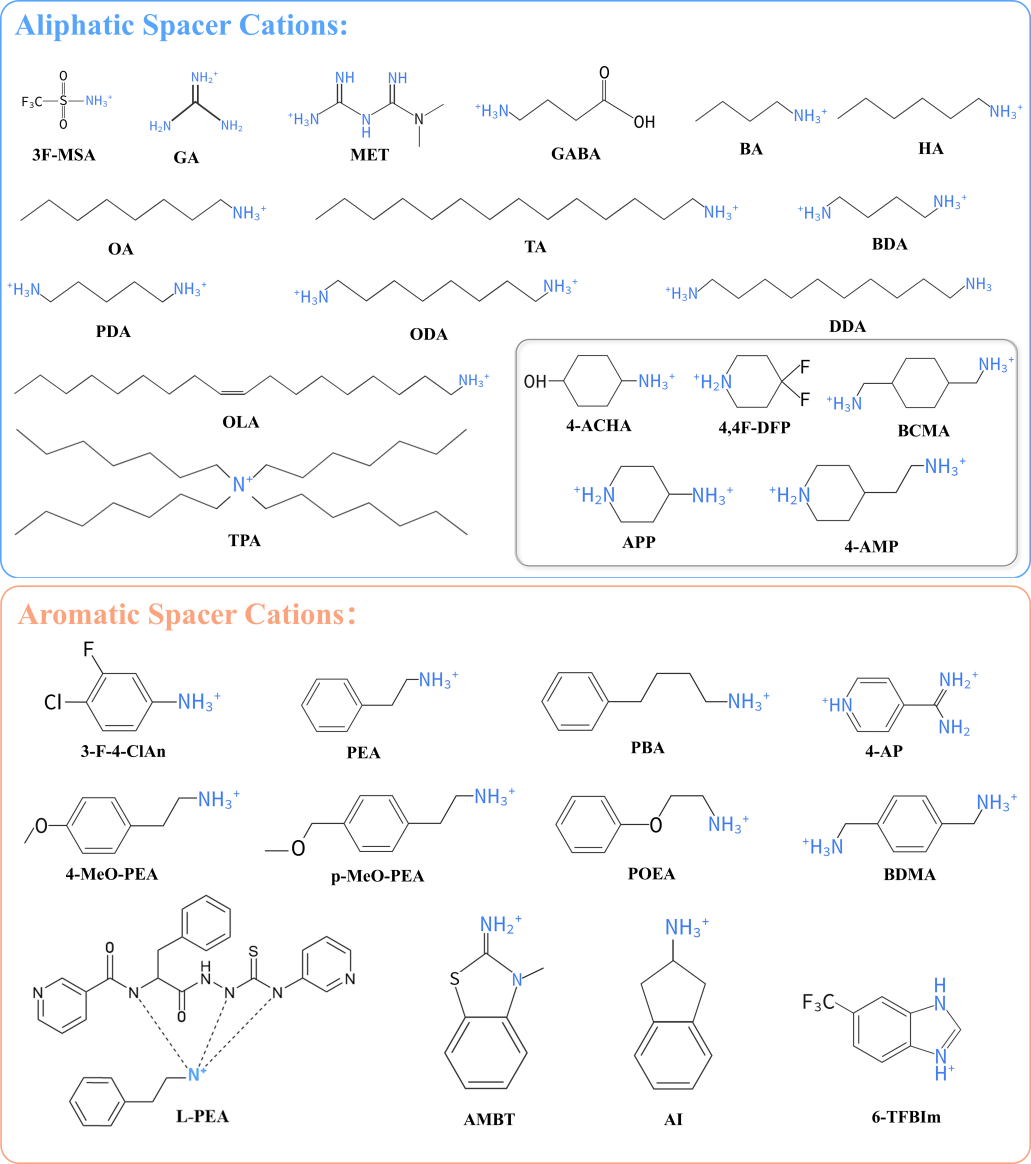
Representative aliphatic (blue) and aromatic (orange) spacer cations used in 2D/3D heterojunctions. Their molecular structures govern quantum-well width, dielectric confinement, and interlayer coupling, thereby modulating carrier mobility, exciton binding energy, and the trade-off between device stability and charge-transport efficiency.

Unlike homojunctions and graded junctions, 2D/3D heterojunction engineering focuses on the rapid reconstruction of electrostatic and energy levels at the interface, thereby in situ enhancing and extending the BEF into the near-interface bulk region [58]. Although isolated 2D (or quasi-2D) phases exhibit excellent chemical stability and defect passivation capabilities, their wide bandgaps and the insulating nature of organic spacer layers often limit charge transport. The organic spacer molecules used in the 2D/3D heterojunctions discussed in this paper are illustrated in Figure 3. Coupling these 2D phases with a 3D bulk to form 2D/3D heterojunctions can, on one hand, establish or extend the BEF via interfacial defect passivation and energy-level cascading, and, on the other hand, retain the high carrier mobility and strong light absorption of the 3D bulk, which has made this approach a research focus in recent years [59–60]. However, the effectiveness of this strategy depends critically on interfacial stability and order. Inappropriate solvent or processing conditions can induce dissolution and recrystallization of the 3D lattice, resulting in energy-level disorder and disruption of the BEF [61–62]. Excessive deposition or a disordered distribution of the low-dimensional phase can further impede carrier transport and may even cause band misalignment, weakening the continuity and depth of the BEF. To address these challenges, various interfacial stabilization strategies have been proposed, aimed at achieving orderly 2D coverage while preserving the integrity of the 3D lattice, thereby establishing a stable and continuous BEF. These efforts have partially mitigated interfacial energy-level disorder and transport limitations, laying a foundation for further enhancing the magnitude and spatial controllability of the BEF.

Conventional alcohol-based treatments, such as isopropanol, can disturb the A-site composition on the surface of 3D perovskites during the formation of 2D/3D heterojunctions, inducing local dissolution and recrystallization, which leads to energy level disorder and phase instability. Surface planarization can mitigate these effects by removing amorphous phases and optimizing energy alignment, but highly soluble solutions may still damage the 3D lattice and introduce defects [63–65]. To balance surface planarization with lattice preservation, Wang et al. [66] employed the long-chain ammonium salt decane-1,10-diammonium diiodide (DDADI) in an acetonitrile (ACN)-based 2D precursor, enabling surface-limited phase conversion, while PbI_2_-assisted phase control (PAPC) suppresses excessive reactions and high-barrier *n* = 1 RP phase formation, promoting a uniform distribution of low-barrier *n* = 2 phases. This synergistic phase control optimizes interfacial energy alignment and charge-transport pathways, enhancing carrier separation, quasi-Fermi-level splitting, and device performance (*V*_oc_ = 1.19 V; *J*_sc_ = 25.59 mA·cm^−2^; PCE = 25.16%). The BEF improves carrier dynamics by reducing trap-assisted recombination and enhancing directional transport, but it can decay over time due to ionic migration and phase segregation. The Dion–Jacobson (DJ)-derived 2D layer and PAPC-controlled *n* = 2 phase stabilize the heterojunction, preserving the BEF under operational stress; the device retained 92.9% of its initial efficiency after 1000 h in nitrogen, and 86.2% under continuous illumination for 500 h.

Although ACN exhibits higher polarity than IPA, it is a polar aprotic solvent with weak Lewis basicity toward Pb^2+^. Consequently, its interaction with the Pb–I framework is more controllable, enabling a relatively mild interfacial modulation. In the half-precursor (HP) approach, ACN is commonly used as a carrier for organic ammonium salts only. These salts undergo ion exchange limited to the surface of the underlying 3D perovskite to form a 2D phase. Without such localized control, 2D spacer cations may penetrate excessively into the bulk perovskite, causing energy level misalignment [67]. By contrast, in the full-precursor strategy, ACN is typically combined with small amounts of strongly coordinating solvents, such as dimethyl sulfoxide (DMSO) or dimethylformamide. This enables codeposition of metal halides and organic ammonium salts, thereby improving phase purity and reducing perturbation to the 3D pervoskite layer [67–68]. However, controlling both the penetration depth and uniformity of the 2D phase remains challenging, particularly in conventional IPA systems. Shan et al. [69] introduced trace amounts of DMSO into the IPA system. DMSO selectively coordinates with surface Pb–I clusters, inducing a transient swelling that lowers the diffusion barrier for 2D spacer cations, promotes subsurface penetration, and facilitates secondary surface crystallization, resulting in a continuous, orientationally ordered *n* = 2 2D layer. This process establishes a stepwise energy level alignment from 3D to 2D, enhancing both the directionality and effective range of *V*_bi_ while ensuring full surface coverage and strong interfacial binding. The DFP-MIX (DMSO-assisted) treatment passivates surface defects (carrier lifetime increased from 29.3 to 115.6 ns), optimizes interfacial energy alignment and contact, and enhances carrier transport efficiency (*V*_oc_ = 1.18 V, FF = 85%) through reduced trap-assisted recombination and improved directional transport. DMSO diffusion further promotes deep penetration of 2D spacer cations and preferential *n* = 2 phase formation, achieving synergistic optimization of defect passivation and charge extraction. Consequently, packaged devices retain 90% of their initial efficiency after 200 h of continuous maximum-power-point operation, whereas reference devices decay below 60%.

Unlike the previously described strategies that rely on polar solvents to assist diffusion for fine control, Wang et al. [70] proposed an interfacial engineering approach based on the nonpolar yet soluble 2D perovskite TPA_2_PbI. This method employs the relatively environmentally friendly and mild solvent ethyl acetate (EtOAc) as the precursor medium, enabling construction of a structurally intact 2D/3D heterojunction interface without disrupting the perovskite lattice. The strategy avoids the need for highly polar solvents to induce diffusion or recrystallization, thereby preventing dissolution of the quasi-2D layer and damage to the 3D perovskite lattice. The resulting 2D layer exhibits a shallow valence band and an elevated Fermi level, which together induce an upward vacuum level shift and band bending at the interface, establishing a strong BEF directed from the 3D to the 2D phase and producing a type-II band alignment. Compared with ACN or DMSO systems, which often struggle to balance diffusion depth and crystal integrity, this approach achieves superior interfacial energy level alignment and spatial continuity of the BEF. Functionally, the 2D layer injects electrons into the 3D surface via chemical bonding, effectively passivating surface defects and reducing trap-assisted recombination. Simultaneously, the type-II alignment generates a directional internal electric field that enhances photogenerated carrier separation and transport. Nonpolar solvent processing combined with the soft lattice of the 2D layer ensures continuous, high-quality coverage, coordinating defect passivation and carrier transport. As a result, the 2D/3D heterojunction extends the carrier lifetime from 29.3 to 115.6 ns, reduces the hole trap density by approximately 17%, increases the fill factor from 82.6% to 84.6%, and achieves a device efficiency of 25.42%. FAPbI_3_-based devices demonstrate exceptional stability, maintaining 96.5% of initial efficiency under dark storage for 2400 h at 80% ± 5% relative humidity, 92.1% under continuous illumination for 1000 h, and 78.4% after 700 h at 60 °C.

To circumvent issues associated with the solution-based fabrication of 2D/3D perovskite heterostructures, such as solvent-induced erosion, interdiffusion of components, and interface mixing, recent strategies have focused on transfer-imprinting-assisted growth and hybrid deposition approaches [71–73]. Lee et al. [74] proposed a solid-state in-plane growth process, in which solid (BA)_2_PbBr_4_ powder is deposited onto the surface of a 3D perovskite and then undergoes low-temperature solid-state diffusion. This method produces a solvent-free, compositionally uniform 3D/2D heterojunction interface. The process yields continuous 2D layers with tunable thicknesses (from 26.5 to 40.1 nm), mitigating interface roughness and compositional inhomogeneity commonly observed in conventional solid-state or solution-processing methods. Compared to iodide-based 2D layers, bromide (BA)_2_PbBr_4_ deepens the 2D work function, shifting *V*_bi_ toward the 3D absorber and optimizing carrier transport. Mechanistically, the 2D coating passivates surface traps, suppresses well-state capture, and guides the internal electric field into the 3D layer, promoting directional separation and extraction of photogenerated carriers. Devices fabricated using this approach achieved a power conversion efficiency of 25.47%, extended carrier lifetime from 2150 to 3990 ns, reduced interface defect density from 1.92 × 10^16^ to 1.03 × 10^16^ cm^−3^, and increased *V*_bi_ from 1.081 to 1.207 V, while retaining 78.4% of the initial PCE after 700 h of thermal aging at 60 °C.

The transition from 3D perovskite to low-n 2D phases requires precise control over electronic properties and band alignment. However, conventional large-molecule incorporation methods often result in disordered vertical distribution of the 2D/3D phases and interfacial disorder, thereby weakening the formation of the BEF [75–76]. Long et al. [77] proposed a pre-embedded junction engineering strategy, in which 1,4-benzenedimethylammonium (BDMA) is pre-introduced into the PbI_2_ framework to directionally induce vertically oriented *n* = 1 DJ phases near the electron transport layer (SnO_2_). This ordered bottom 2D layer simultaneously passivates interfacial defects, reducing trap density from 6.30 × 10^15^ cm^−3^ to 3.15 × 10^15^ cm^−3^, and optimizes interfacial energy-level alignment, lowering carrier transport barriers. Consequently, the BEF is enhanced, and directional charge separation is promoted. Mechanistically, BDMA passivates deep-level defects, particularly undercoordinated Pb^2+^, converting them into shallow states to suppress trap-assisted recombination, while the buried 2D/3D interface modifies band alignment and redistributes the BEF into the 3D absorber, driving efficient directional carrier extraction. The combined effect of defect passivation and BEF-guided carrier transport accounts for improved carrier lifetime, charge extraction, and device stability. BDMA-modified perovskites retained structural integrity after 1080 h under 50–60% RH, 216 h at 60 °C, and 1050 h of continuous illumination, maintaining 88–97% of initial device performance, whereas unmodified perovskites degraded within, respectively, 360 and 120 h, or under similar light exposure. Ultimately, this orientation-induced configuration increased device efficiency from 20.64% to 23.40% and highlights the synergistic role of interfacial stabilization and BEF enhancement.

In the exploration of the multi-interface synergistic enhancement of *V*_bi_, previous studies have simultaneously optimized the top and bottom 2D/3D interfaces and have incorporated structural confinement and dipole engineering to improve energy-level alignment and interfacial stability. Xiong et al. [78] proposed a bidirectional interface optimization strategy: Seed layers of BAMAPbI (*n* = 3, 4, 5) were spin-coated onto the hole transport layer (MeO-2PACz) to form a bottom 2D induction layer, promoting the epitaxial crystallization of the overlying 3D perovskite and improving interfacial contact, thereby enhancing hole extraction and reducing thermal emission losses. Subsequently, a 4-methoxyphenylphosphonic acid (MPA) dipole passivation layer was introduced onto the surface of the 3D perovskite, followed by the deposition of the top 2D layer, forming a three-tiered configuration of HTL/2D/3D/MPA/2D/ETL (where the ETL is PCBM). Serving as a dipole layer, MPA minimizes the energy-level offset at the top 3D/2D interface, and, combined with the cascade alignment established by the bottom 2D layer, yields a more continuous vertical potential profile that strengthens the BEF. Within this framework, crystallographic ordering and bulk defect suppression originate predominantly from bottom 2D-guided epitaxy, whereas the top MPA/2D complex contributes mainly to dipole-driven band reconstruction and interfacial passivation. Consequently, the dual 2D/3D/2D interfaces jointly suppress trap-assisted recombination and reinforce directional charge transport through an enhanced BEF, enabling a champion PCE of 25.25%. Stability is likewise improved, with the unencapsulated device retaining 95% of its initial efficiency after 1200 h in 65% relative humidity and 90% after 900 h under continuous 1-sun illumination in nitrogen.

Existing 2D/3D heterojunction strategies have largely focused on surface passivation [79–80], often overlooking compositional and lattice inhomogeneities within the perovskite bulk [81–82]. These bulk inhomogeneities induce tensile strain and generate deep traps that weaken the BEF and accelerate nonradiative recombination [83–84]. Hou et al. [85] proposed the bulk in situ reconstruction strategy, in which a diammonium cation with a cyclohexane backbone (BCMA^2+^) was added to the PbI_2_ precursor. These cations self-assemble into DJ-type 2D nuclei that are uniformly distributed throughout the film, inducing in situ growth of the 3D phase across the film thickness and constructing a gradient 2D/3D heterostructure that spans the entire film. Owing to their flexible molecular conformation, these nuclei buffer lattice tensile strain, promote ordered crystallization through strong 2D/3D interfacial interactions, and suppress bulk defect formation. This structural reconstruction also shifts the valence band maximum from 5.70 to 5.56 eV, establishing a favorable type-II cascade alignment that strengthens the vertical BEF. The resulting graded junction exhibits a 66.6% reduction in trap density and a 43% decrease in carrier lifetime, indicating accelerated extraction and suppressed SRH recombination, with electrical measurements confirming the enhancement of *V*_bi_ and the efficiency of directional transport. Under 35% RH, the device maintained 76.2% of its initial efficiency after 3220 h (vs 44.9% for the control), and the perovskite film showed no detectable chemical or structural degradation after 120 days, demonstrating that bulk-embedded graded junction engineering effectively couples strain release, defect suppression, and band-edge modulation to reinforce the BEF and yield high stability and efficiency in perovskite solar cells.

#### Dipole and polarization engineering for BEF enhancement

In perovskite solar cells, beyond interfacial modification and energy level alignment used to control the potential distribution, polarization effects play an important role in enhancing the BEF [86]. Polarization fields introduce additional internal potential gradients within the material, thereby increasing the driving force for charge carrier separation and transport [87]. They also help mitigate common failure mechanisms such as nonradiative recombination and ion migration. However, the practical impact of polarization is often limited by disorder in dipole orientation, unstable polarization domains, and ionic screening. These factors can cause the field enhancement to decay or become spatially nonuniform. To address these challenges, researchers have proposed several strategies, including modifying interfaces with polar molecules whose orientation can be controlled, introducing intrinsically polar low-dimensional perovskites or other 2D materials, and inducing bulk polarization through strain and symmetry breaking. All strategies aim to establish stable, persistent polarization potential profiles across the film thickness. These efforts open new avenues to further increase the magnitude of the BEF and to achieve finer spatial control.

In 2D/3D heterojunctions, mismatches in conduction type or interfacial work function (ΔΦ) between the 2D layer and 3D matrix limit band bending and weaken the BEF, reducing the driving force for carrier separation and transport. Previous studies of molecular dipole modulation have largely focused on short-chain organic ammonium cations, neglecting systematic exploration of chain-length effects, particularly in HTL-free architectures where the interface must simultaneously passivate defects, align energy levels, and provide sufficient *V*_bi_. Tang et al. [88] conducted a comprehensive study of linear alkylammonium cation chain length, spanning BAI to ODAI, and demonstrated that as chain length and dipole moment increase, the 2D layer gradually converts from n-type to weakly p-type, amplifying the interfacial work function difference and enhancing the potential gradient toward the carbon electrode [89]. This dual effect simultaneously improves carrier separation and suppresses trap-assisted recombination, while excessive chain length introduces an insulating barrier that impedes charge transport. Balancing defect passivation and directional transport, heterojunctions formed with tetradecylammonium cations (TA^+^, C14) achieve optimal BEF enhancement: VTFL decreases from 0.177 to 0.114 V, charge transport time shortens from 1.44 to 0.57 μs, and *V*_bi_ is increased. The resulting planar HTL-free carbon PSC reaches a PCE of 20.40% (certified 20.1%) with excellent operational stability (900 h MPP retention 95.9%; 1500 h at 25 °C/50% RH only 4.1% attenuation). The core mechanism lies in the formation of a p-type 2D layer via long-chain cations, which constructs a strong BEF while simultaneously blocking water and oxygen permeation channels, thereby optimizing carrier dynamics and device longevity.

In addition to optimizing interfacial potential by adjusting the alkylammonium chain length and molecular dipole moment, modifying the molecular structure is another effective strategy for improving 2D/3D heterojunction performance. For instance, aromatic spacer cations (e.g., phenethylammonium (PEA^+^) [90], pyridine-based and indene-type derivatives [91–92], and sulfur-or nitrogen-containing heteroaromatic derivatives [93]) take advantage of π-conjugation and relatively large molecular dipoles. These cations often promote preferential film orientation through π–π stacking and hydrogen bonding or coordination interactions with the perovskite framework, improving interfacial band alignment and charge-carrier separation efficiency. Building on this, Deng et al. [94] demonstrated that a resonance-stabilized iminium-type spacer cation (AMBTI) forms a flat *n* = 1 2D layer [(AMBT)_2_PbI_4_] on FA_0.88_Cs_0.12_PbI_3_, which enhances BEF via resonant charge delocalization and molecular polarization, effectively passivates defects, increases carrier lifetime, and reduces defect density by 44.4%. These improvements translate into enhanced *V*_oc_ and FF, yielding a champion PCE of 24.2% (*V*_oc_ = 1.166 V, FF = 81.36%), along with excellent stability (>90% PCE retention after 500 h at 25 °C and 40% RH). In contrast, 1D spacer cations (AMT^+^) form sharp needle-like crystals that introduce interfacial capacitance and charge leakage, disrupt directional transport, and significantly degrade device performance. The synergistic effect of the hydrophobic 2D layer and the interfacial potential build-up suppresses water- and oxygen-induced PbI_2_ formation and phase separation, further contributing to the long-term operational stability.

In addition to resonant charge delocalization, dipole enhancement can also be achieved by introducing large organic cations bearing multiple halogen substituents [95]. Compared with conventional monohalogen-substituted structures, molecules substituted with two halogen atoms demonstrate higher molecular polarity and a stronger ability to induce orientational ordering. These characteristics can markedly improve the ordering of the 2D layer and enhance interfacial polarization effects. Du et al. [96] introduced dual-halogen-substituted *meta*- and *para*-aminophenyl iodides (e.g., 3-F-4-ClAnI and 3-Cl-4-FAnI) into a CsMAFAPb(IBr) precursor. This treatment induced the formation of *n* = 2 RP phase two-dimensional perovskites that templated the preferential epitaxial growth of the three-dimensional perovskite along the same crystallographic direction. Among the compounds examined, 3-F-4-ClAnI-derived heterostructures exhibited the highest 2D layer ordering and interfacial matching, indicating that increased molecular polarity and the spatial configuration (e.g., cis/trans isomerism) further amplify interfacial polarization effects. This 2D template not only directs vertical epitaxial growth of the 3D phase but also blocks water and oxygen penetration, ensuring long-term stability. Meanwhile, the BEF is enhanced through a collaborative “passivation + transmission” mechanism: Strong N–H^...^I hydrogen bonding and Pb-halide coordination stabilize the 2D layer for efficient defect passivation, while the ordered 2D layer guides 3D epitaxial growth, optimizes band alignment, and improves directional carrier transport, ultimately enabling a power conversion efficiency of 24.74% and maintaining 91.6% and 83.6% of initial efficiency under 25 °C, 50% RH dark state and continuous LED illumination in N_2_ for 1000 h, respectively.

**Figure 4:**
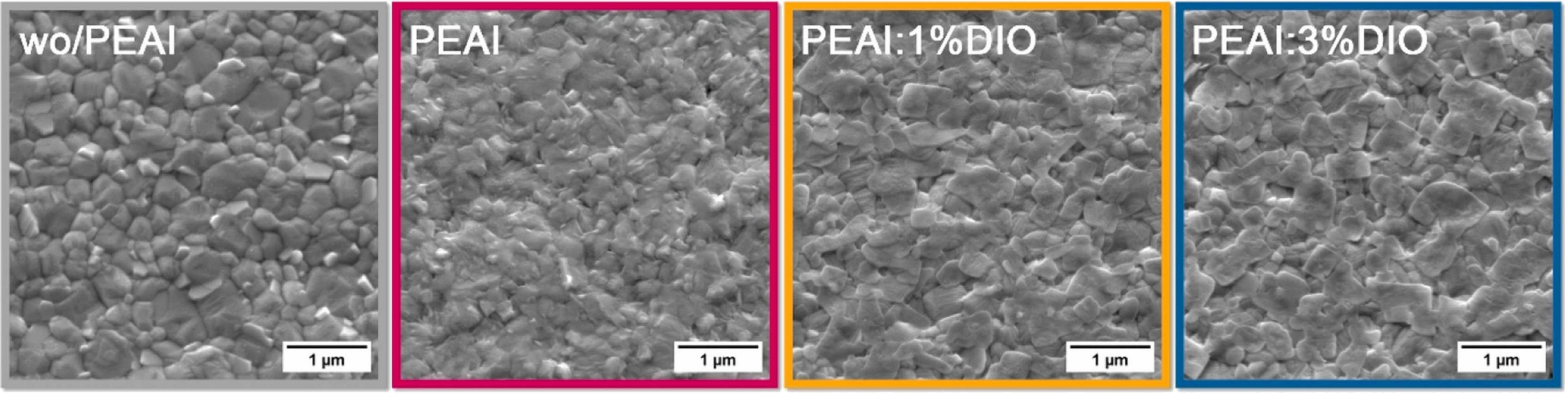
Top-view scanning electron microscopy (SEM) images (scale bar: 1 μm) of CsFAMA perovskite films under different passivation conditions. The PEAI:DIO synergistic treatment effectively promotes larger grain size, higher crystallinity, and more compact film morphology, which helps reduce defect states and improve charge transport in perovskite devices. Figure 4 was reproduced from [100] (© 2025 F. L. de Araujo et al. Published by Elsevier Ltd., distributed under the terms of the Creative Commons Attribution 4.0 International License, https://creativecommons.org/licenses/by/4.0/).

In two-dimensional perovskite heterojunctions, the interfacial molecular orientation in some systems is poorly ordered, and the polarization directions are randomly dispersed, resulting in a reduced contribution of dipole layers to the BEF. The DJ phase, based on hydrogen-bonded diammonium cations, provides excellent stability and carrier transport, but its slow crystallization kinetics and high molecular symmetry limit the formation of consistently oriented polarization layers; while long- or short-chain diammonium modifications (e.g., ODA, PDAD, APP) improve stability [97–99], polarization enhancement remains less effective than in monoammonium RP systems. De Araujo et al. [100] demonstrated that spin-coating a mixed solution of DIO and PEA^+^ produces an ordered surface structure at room temperature, promoting long-range grain ordering and surface-directed enrichment of PEA^+^ to form an in-plane oriented dipole layer (see Figure 4), which increases *V*_bi_ from 1.08 to 1.15 V and raises the surface work function from 4.72 to 4.97 eV, as confirmed by KPFM measurements. The PEAI:DIO synergistic treatment enhances the BEF through two pathways: DIO and PEAI cooperatively passivate surface defects, reducing trap-mediated recombination, while DIO-induced interface dipoles optimize band alignment and directional carrier transport, resulting in an 8.6% increase in carrier lifetime, a fill factor of 80.00%, and a PCE of 20.25%. At the same time, the formation of a high-quality *n* = 1 2D layer inhibits ion migration and blocks water and oxygen penetration, maintaining 80% of initial efficiency after 1600 h at 55% RH and 25 °C, achieving a threefold increase in T80 lifetime.

Although two-dimensional layers based on monoammonium cations can generate dipole fields at the surface, their effect is largely confined to local interfacial regions, which limits the modulation of the overall BEF. Zhang et al. [101] introduced 4-MeO-PEAI and n-HeXAI into a mixed antisolvent system to induce the formation of a highly (001)-out-of-plane-oriented 2D/3D heterojunction with a thin and uniform 2D capping layer, which improves crystal orientation and facilitates vertical carrier transport. This structure substantially enhances interfacial band bending and barrier gradients, with Mott–Schottky analysis revealing an increase in *V*_bi_ from 0.81 V in the control sample to 0.92 V in the target device, indicating a significant augmentation of the device’s internal potential. UPS and KPFM further confirmed the polarization-induced shifts in work function and the enhancement of the built-in potential. The resulting dual-path optimization combines 2D capping-layer passivation of interface defects, which reduces trap-assisted recombination, with long-chain ammonium salt-induced (001) crystal-plane orientation, which improves vertical charge transport. These synergistic effects increased carrier lifetime to 96.2 ns, reduced the ideal factor to 1.3, raised the fill factor to 85.12%, and enabled a PCE of 26.02% (certified 25.42%), while also providing outstanding stability: The device retained 87.6% efficiency after 7000 h of dark storage (ISOS-D-1) and 97.8% after 2100 h of continuous light MPP tracking (ISOS-L-2), with an extrapolated T80 lifetime exceeding 10000 h, demonstrating the combined benefits of interface passivation and oriented crystal growth in enhancing both performance and long-term stability.

Several studies have further extended the polarization effect from local interfaces vertically through perovskite films, with the aim of enhancing the BEF across the entire film thickness. Ran et al. [102] employed a dual-path protonation strategy, using either acidification with HI or treatment with DMBG hydrochloride, to locally convert perovskite grain boundaries into vertically oriented two-dimensional layers with spontaneous polarization. This conversion markedly increased both the depth and the magnitude of the BEF. Protonation significantly increased the interfacial work-function difference and the surface potential contrast, which enhanced band bending and thereby strengthened the potential gradient across the film thickness. The resulting energy-level staircase structure, together with the enhanced BEF, effectively reduced the hole-extraction barrier and suppressed interfacial recombination under conditions of anisotropic conductivity [103]. The modulation of carrier dynamics by the enhanced BEF was further confirmed through TRPL, which revealed a reduction in trap-state density and an increase in carrier lifetime. This approach produced a PSC of 22.09% and yielded favorable performance in C-PSCs. Protonation, by adjusting the p*K*_a_ difference, induced the vertical alignment of *n* = 1 2D perovskite at the grain boundaries, thus blocking the MA^+^ volatilization pathway and preventing the Pb^0^/δ-PbI_2_ phase transition. Under nitrogen conditions, the device maintained 98.5% PCE after 720 h, while under ambient conditions (25 °C, 35% RH), no degradation of the MA peak was observed after 336 h. The core mechanism behind this stability is the vertically oriented 2D perovskite layers, which act as barriers to ion migration and moisture/oxygen infiltration.

To further extend the spatial scope of polarization effects, Tang et al. [104] introduced a penetrating 2D/3D heterojunction interlinked (ILDH) strategy. Through the synergistic infiltration of POEA^+^ and SCN^−^ at grain boundaries and dual interfaces, quasi-2D layers form a gradient distribution along the 3D grain boundaries, thereby establishing a continuous polarization field vertically through the film. This architecture not only extends the interfacial-dipole effect into the film bulk but also provides low-resistance electron-transport channels through the exposed Pb–I framework, thereby amplifying and homogenizing the spatial distribution of the BEF. Transient spectroscopic and electrical analyses further revealed two major carrier-dynamic enhancement pathways: SCN^−^-assisted 2D perovskite encapsulation effectively passivates interface and grain-boundary defects, significantly reducing trap-state capture. Meanwhile, the graded quasi-2D distribution reconstructs local band bending, optimizes band alignment, and reshapes the internal electric-field landscape, alleviating electron-transport barriers characteristic of planar type-II interfaces. Benefiting from these synergistic mechanisms, ILDH devices achieve certified efficiencies above 24%, with the POEAI-NH_4_SCN device reaching 24.76% and retaining over 90% of its initial efficiency after 800 h under 85 °C heating or 65% humidity, owing to combined effects of electric-field optimization, stress relaxation, and grain-boundary sealing.

In 2D/3D heterojunction perovskite systems, maintaining a stable and long-lasting BEF is more complex due to the intricate nature of the interface. Unlike homojunctions, which rely on relatively simple band bending, and gradient junctions, which use compositional gradients, 2D/3D heterojunctions are highly sensitive to interface instability, phase segregation, and ion migration. These factors can cause degradation pathways, including the flattening of the BEF, misalignment of energy levels, and increased recombination rates, especially under thermal and humidity stress [105]. To mitigate these issues, strategies such as using non-polar solvents like ethyl acetate to prevent lattice dissolution, and long-chain organic ammonium salts (e.g., DDADI) for uniform 2D coverage, are essential. These approaches help preserve interface integrity and maintain a well-aligned energy landscape.

Compared to homojunctions, which produce relatively shallow BEFs, and gradient junctions, which are prone to ion-induced flattening of the BEF, 2D/3D heterojunctions offer more localized and robust BEFs, with superior interfacial defect control. However, their processing sensitivity remains a significant challenge for large-scale fabrication. To improve stability and performance, additional stabilization strategies, including ion-blocking layers (e.g., Al_2_O_3_), precise control over 2D phase orientation, and the use of hydrophobic materials or stress-buffering components, can help preserve BEF continuity and minimize environmentally driven degradation. By integrating these methods, 2D/3D heterojunctions can complement the strengths of homojunctions and gradient junctions, offering a promising path for more stable and scalable perovskite photovoltaics.

#### Comparative analysis of junction strategies for BEF enhancement

Homojunctions, gradient junctions, and 2D/3D heterojunctions modulate the BEF via fundamentally different mechanisms, leading to unique performance advantages and constraints when applied at different scales and in real-world device architectures.

Homojunctions create localized band bending via carrier-density variations within a single perovskite phase. Their minimal structural discontinuity and ease of solution processing make them highly manufacturable, with potential for low-cost, large-area fabrication. However, the resulting BEF is typically shallow and sensitive to factors such as dopant uniformity, defect evolution, and ion migration, which can degrade the BEF over time. These limitations restrict their long-term stability, particularly in high-performance applications.

Gradient junctions introduce compositional or energetic transitions that establish continuous potential slopes across the film thickness, which extends the BEF deeper into the material and improves bulk carrier extraction. This strategy is particularly beneficial for wide-bandgap and tandem device architectures, where the enhancement of the BEF can boost performance significantly. However, maintaining the uniformity of the gradient requires precise control over diffusion, crystallization, and ion redistribution. In practice, these factors may lead to non-ideal gradients, flattening the BEF over time and reducing the effectiveness of the device.

2D/3D heterojunctions enhance BEF by generating strong interfacial fields through dipole formation and polarization-assisted band alignment [106]. These interfacial fields significantly improve *V*_oc_ and device stability by mitigating recombination losses and enhancing energy-level alignment. However, achieving consistent performance in 2D/3D heterojunctions requires careful control over 2D layer coverage, phase penetration, and solvent–crystal interactions. These factors make it difficult to scale up this technology for large-area processing, especially when dealing with non-uniform or unstable interfaces.

To understand and optimize the influence of the BEF, studies should focus on its direct regulation of carrier dynamics. BEF can enhance directional charge transport and suppress trap-state capture, thereby improving carrier lifetime and mobility. First-principles calculations of band alignment and interfacial dipole distribution, combined with molecular dynamics simulations and machine learning techniques, can guide experimental design and provide a rigorous validation of the proposed mechanisms. By correlating theoretical predictions with experimental BEF parameters and energy-level alignment, as outlined in Table 3, where we have maximized the selection of representative strategies for a comprehensive comparison, we can quantitatively understand how junction design governs charge separation, transport, and recombination. This approach offers a robust framework for optimizing BEF and improving device performance.

**Table 3:** Quantitative comparison of representative BEF enhancement strategies in perovskite solar cells.

Junction type	Strategy	*V*_bi_	Test method	Electric field indicator	Test technique	BEF depth	PCE improvement	Ref.

p/p^+^ homojunction	Ag-doped NiO*_x_*	0.09 V	*J*–*V* characterization	ΔWF = 0.09 eV	UPS	about 30 nm	18.83% (16.20% → 19.25%)	[30]
p/n homojunction	anion-assisted molecular doping	significantly enlarged	Mott–Schottky	ΔWF = 0.45 eV	KPFM	N/A	18.7% (20.48% → 23.89%)	[31]
n/p homojunction	vitamin-enabled homojunction	1.058 V	Mott–Schottky	ΔWF = 0.53 eV	UPS	80 nm	6.75% (22.67% → 24.20%)	[37]
n/p homojunction	black-phosphorus-enabled homojunction	0.97 V	Mott–Schottky	ΔWF = 0.24 eV	UPS	80 nm	11.90% (20.75% → 23.22%)	[38]
gradient junction	bottom-up ionic gradient	0.81 V	Mott–Schottky	Δ*E* = 0.04 eV	KPFM, GIXRD	N/A	2.67% (8.75% → 11.42%)	[45]
gradient junction	synergistic defect-energy optimization	1.14 V	Mott–Schottky	ΔVBM = 0.33 eV	UPS, KPFM	N/A	1.55% (16.22% → 17.77%)	[57]
2D/3D heterojunction	n-value control	1.001 V	Mott–Schottky	HOMO level upshift	UPS	N/A	2.53% (22.63% → 25.16%)	[66]
2D/3D heterojunction	work-function-enhanced junctions	1.150 V	Mott–Schottky	ΔWF = 0.34 eV	UPS, KPFM	N/A	3.17% (22.30% → 25.47%)	[74]
2D/3D heterojunction	anti-solvent orientation control	0.92 V	Mott–Schottky	ΔCPD = −58.88 mV	KPFM	about 10 nm	1.93% (24.09% → 26.02%)	[101]

Rather than being mutually exclusive, homojunctions, gradient junctions, and 2D/3D heterojunctions can complement each other when integrated into hybrid architectures. By combining carrier-density tuning, compositional gradients, and interfacial dipoles, hybrid strategies hold the potential to maximize BEF magnitude, spatial coherence, and long-term device stability. These hybrid approaches leverage the strengths of each strategy while mitigating their individual limitations, offering a promising pathway for next-generation high-efficiency perovskite solar cells.

## Conclusion

This review summarizes recent advances in junction engineering strategies, including homojunctions, gradient junctions, and 2D/3D heterojunctions, for enhancing the built-in electric field in perovskite solar cells. These strategies regulate energy-level alignment, induce interfacial dipoles, or construct vertical gradients to promote carrier separation, suppress non-radiative recombination, and improve device efficiency and stability.

Future progress will center on strategies that integrate material, molecular, and fabrication control to achieve deeper, more stable, and spatially coherent BEF modulation. Emerging perovskite derivatives with tunable band structures, such as halide double perovskites, enable wider bandgap gradients and more uniform field distribution. Their combination with low-dimensional or polarizable components can amplify local field strength, while ferroelectric interfaces offer persistent polarization for long-range band bending.

Parallel to material development, molecular design with large dipole moments provides a direct route to strengthen interfacial polarization while passivating defects. Achieving these tailored junctions requires precise and non-destructive fabrication methods; techniques such as solid-phase epitaxy or vapor-phase conversion allow for controlled 2D/3D interface formation and well-defined vertical gradients with minimal lattice perturbation.

Overall, the next phase of junction engineering will be defined by quantitatively guided BEF design, depth-spanning hybrid architectures, and scalable fabrication routes. Integrating strong dipolar molecules at 2D/3D interfaces or combining wide-bandgap and narrow-bandgap phases within gradient junctions can extend field penetration, broaden spectral response, and enhance charge transport. Advancements in these areas will collectively enable stronger and more stable BEF, leading to higher open-circuit voltage, improved fill factor, and longer-lived perovskite photovoltaics.

## Data Availability

Data sharing is not applicable as no new data was generated or analyzed in this study.
